# Type I Interferon Programs Innate Myeloid Dynamics and Gene Expression in the Virally Infected Nervous System

**DOI:** 10.1371/journal.ppat.1003395

**Published:** 2013-05-30

**Authors:** Debasis Nayak, Kory R. Johnson, Sara Heydari, Theodore L. Roth, Bernd H. Zinselmeyer, Dorian B. McGavern

**Affiliations:** National Institute of Neurological Disorders and Stroke, National Institutes of Health, Bethesda, Maryland, United States of America; The Fox Chase Cancer Center, United States of America

## Abstract

Viral infections of central nervous system (CNS) often trigger inflammatory responses that give rise to a wide range of pathological outcomes. The CNS is equipped with an elaborate network of innate immune sentinels (e.g. microglia, macrophages, dendritic cells) that routinely serve as first responders to these infections. The mechanisms that underlie the dynamic programming of these cells following CNS viral infection remain undefined. To gain insights into this programming, we utilized a combination of genomic and two-photon imaging approaches to study a pure innate immune response to a noncytopathic virus (lymphocytic choriomeningitis virus) as it established persistence in the brain. This enabled us to evaluate how global gene expression patterns were translated into myeloid cell dynamics following infection. Two-photon imaging studies revealed that innate myeloid cells mounted a vigorous early response to viral infection characterized by enhanced vascular patrolling and a complete morphological transformation. Interestingly, innate immune activity subsided over time and returned to a quasi-normal state as the virus established widespread persistence in the brain. At the genomic level, early myeloid cell dynamics were associated with massive changes in CNS gene expression, most of which declined over time and were linked to type I interferon signaling (IFN-I). Surprisingly, in the absence of IFN-I signaling, almost no differential gene expression was observed in the nervous system despite increased viral loads. In addition, two-photon imaging studies revealed that IFN-I receptor deficient myeloid cells were unresponsive to viral infection and remained in a naïve state. These data demonstrate that IFN-I engages non-redundant programming responsible for nearly all innate immune activity in the brain following a noncytopathic viral infection. This Achilles' heel could explain why so many neurotropic viruses have acquired strategies to suppress IFN-I.

## Author Summary

The central nervous system is equipped with innate immune cells that serve as first responders to sterile injuries and infections. The mechanisms that program the movement and morphological transformations of these cells following infection remain undefined. Here, we utilized a combination of genomic and *in vivo* imaging approaches to define pathways that program the motion of innate immune cells responding to a noncytopathic virus as it established persistence in the brain. *In vivo* imaging studies performed in the living brain revealed that innate myeloid cells mounted a vigorous early response that returned to a “naïve” state during persistence. This was associated at the genomic level with robust changes in gene expression that were mostly quenched over time. Analysis of the gene expression pattern revealed a prominent type I interferon (IFN-I) signature only at the early stage of infection. Surprisingly, in the absence of type I interferon (IFN-I) signaling, almost no genes were differentially expressed in the virally infected nervous system and all innate myeloid cells were unresponsive. These data indicate IFN-I programs all innate myeloid activity in the nervous system following a noncytopathic viral infection. This non-redundant anti-viral program represents an Achilles' heel that can be exploited by neurotropic viruses.

## Introduction

The central nervous system (CNS) is an immunologically specialized compartment consisting of the brain and spinal cord [1]. These structures are lined by what is referred to as the meninges. Most blood vessels within the CNS are non-fenestrated, meaning the endothelial cells that comprise these vessels are connected by tight junctions which limit the influx of vascular materials into the CNS [2]. Tight junctions are a key feature of the blood brain and blood cerebral spinal fluid barriers that help protect the CNS from peripheral challenges. Despite this elaborate barrier structure, many infectious agents have evolved clever strategies to access the CNS [3]. This tissue must therefore be equipped to mount an immune response to preserve its cellular inhabitants, some of which are non-replicative (e.g. neurons). Because most immune responses begin with pattern recognition or the sensing of “danger” [4], [5], tissues often possess elaborate networks of innate immune sentinels that typically serve as the first responders to infectious agents. Despite its immunoprivileged status [6], the CNS is no different from the periphery in this regard. The most abundant innate immune sentinels in the CNS are referred to as microglia [7]. These cells are ramified and distributed evenly throughout the CNS parenchyma. In addition, recent intravital imaging studies have demonstrated that microglia processes are highly dynamic and continually scan the CNS [8], [9]. The meninges, choroid plexus, and perivascular spaces in the CNS are also inhabited by specialized macrophages as well as dendritic cells (DCs) [10], [11], [12], [13]. Unlike microglia [14], these cells are hematopoietically-derived and turnover at regular intervals [12]. In fact, a recent study demonstrated that DCs residing in the meninges and choroid plexus are Flt3-ligand responsive and have a 5–7 day half-life [12]. Thus, the innate immune composition of the CNS lining in some ways resembles that observed in peripheral tissues.

Because many infectious agents can invade and replicate within the CNS, it is critical that the innate immune sentinels who inhabit this environment be able to respond quickly and provide the inflammatory cues required to recruit a successful adaptive response. It is also imperative that this be accomplished in a minimally injurious manner so as to preserve the integrity of the nervous system. A key element in most innate anti-viral responses is the production of type I interferons (IFN-I) [15], [16]. There are many different IFN-I subtypes (alpha, beta, delta, epsilon, kappa, omega, tau, zeta), but they all bind to the IFN-α receptor (IFNAR), which consists of two chains (IFNAR1 and IFNAR2). Upon receptor binding, IFN-I can induce an anti-viral state within responding cells [15], [17], [18] and facilitate the development of an adaptive immune response by promoting antigen presentation, immune recruitment, *etc*. The timing and coordination of this response is critical and often dictates that outcome of an infection. In the absence IFN-I, nearly all neurotropic viruses tested acquire a replicative advantage within the CNS and increase their virulence [19]. This explains why so many mammalian viruses have evolved strategies to subvert the IFN-I system [20], [21].

While the importance of innate immunity in protecting the CNS from neurotropic infections is well recognized [19], little is known about how the innate immune program translates into cellular dynamics upon viral challenge. It is also unclear to what degree interferons influence the global responsiveness of the virally infected CNS. We sought mechanistic insights into the dynamics of this process by using the lymphocytic choriomeningitis virus (LCMV) model system. LCMV is a non-cytopathic arenavirus ideally suited to study pure innate immunity. The virus is detected by retinoic acid-inducible gene I (RIG-I) and melanoma-differentiation-associated gene 5 (MDA5) – two cytosolic pathogen recognition receptors [22]. In addition, LCMV does not directly kill the cells it infects *in vivo*, which eliminates a potential confounding variable associated with release of damage associated molecular pattern molecules (DAMPs) from injured cells [23]. Intracerebral inoculation of immunocompetent mice with LCMV results in the development of fatal meningitis at day 6 post-infection, which is due to infiltrating cytotoxic lymphocytes (CTL) and myelomonocytic cells [24]. We previously observed that LCMV establishes lifelong persistence in the CNS of mice with a CD8^+^ T cell repertoire directed against ovalbumin (OT-I mice) [25], [26]. OT-I mice become asymptomatic viral carriers due to their inability to mount either a virus-specific CD8^+^ or CD4^+^ T cell response against LCMV [25]. Following intracerebral inoculation, LCMV first replicates in the meninges of OT-I mice and then moves into parenchymal astrocytes over a two month period [26]. At later stages of viral persistence (4–5 months), LCMV transitions into oligodendrocytes. The reproducibility of this model afforded us the opportunity to study how the brain responds innately to the establishment of a persistent viral infection. Using a combination of genomic and dynamic imaging approaches, we revealed that the brain mounts a vigorous innate response to LCMV that subsides as the virus transitions from the meninges into the parenchyma. Interestingly, we identified an Achilles' heel in the brain's innate defense against this non-cytopathic arenavirus. In the absence of IFN-I signaling, all innate immune gene expression and cellular dynamics were abrogated, which rendered LCMV invisible to its murine host.

## Results

### LCMV tropism shift is not associated with viral mutation

**Figure 1:**
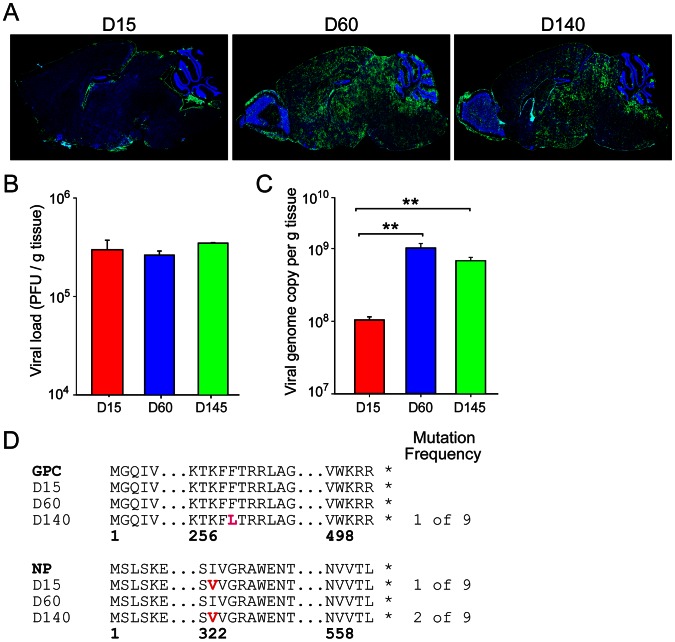
The shift in LCMV tropism during viral persistence is not associated with mutation in viral proteins. (A) Representative sagittal brain reconstructions show the distribution of LCMV (green) at different time points following intracerebral inoculation of OT-I mice (n = 3 mice per group). At early time points post-infection (days 15), LCMV antigen was restricted primarily to the meninges, choroid plexus, and ependyma. By 60 days, the virus spread from the lining into the brain parenchyma, infecting astrocytes primarily. Finally, at late time points post-infection (days 140), the virus shifted tropism and replicated in oligodendrocytes [26]. Cell nuclei are shown in blue. (B, C) Viral titers were quantified in the brains of OT-I mice by plaque assay (B) and Q-PCR (C) at days 15, 60, and 140 post-infection (n = 3 mice per group). Note that the titer of infectious virus as determined by plaque assay was comparable at all time points despite the dramatic change in viral distribution. The number of viral genome copies increased significantly (**, *p*<0.05) as LCMV moved into the brain parenchyma at days 60 and 140 post-infection. (D) Sequences of the LCMV glycoprotein (GPC) and nucleoprotein (NP) were obtained from viral isolates (n = 3 independent isolates) extracted from the brains of OT-I mice (n = 3 mice per time point) at the denoted time points post-infection. Mutational frequencies and the mutated amino acids (red letters) are provided. Data are representative of two independent experiments.

Unlike wild type B6 mice, we previously observed that OT-I mice [27], [28] infected intracerebrally with LCMV do not develop neurological symptoms or fatal meningitis due to an inability to mount an LCMV-specific CD8 or CD4 T cell response [25], [26]. Instead, LCMV establishes lifelong persistence in the brain and peripheral tissues of OT-I mice, and they remain asymptomatic. Over time the virus transitions from the meninges (day 15; Fig. 1A) to the brain parenchyma (days 60 and 140; Fig. 1A). Once within the parenchyma, the virus first replicates in astrocytes (day 60) and later gains access to oligodendrocyes (day 140) [26]. These three time points were therefore selected to represent the different stages of LCMV persistence in a murine host rendered deficient in eliciting an adaptive immune response. To gain insights into the factors associated with LCMV persistence in the nervous system, we first quantified viral loads by plaque assay (Fig. 1B) and quantitative-PCR (Q-PCR) [29] (Fig. 1C) at the denoted time points post-infection. Plaque assay is a measure of infectious virus, and, interestingly, no significant difference in infectious virus was detected at any of the time points (Fig. 1B), despite a major change in viral antigen distribution as demonstrated by immunohistochemistry (Fig. 1A). As virus inundated the brain parenchyma at day 60, a one log increase (*p*<0.05) in viral genome copies was observed by Q-PCR relative to the day 15 time point (Fig. 1C). No significant differences were detected between days 60 and 140. The discrepancy between the viral plaque and Q-PCR results suggest that LCMV produces more defective interfering (non-infectious) particles upon entering the brain parenchyma.

To determine if the shift in LCMV tropism was associated with mutation(s), we next performed sequence analyses on viral clones extracted from the brains of OT-I mice at days 15, 60, and 140 post-infection (Fig. 1D). We sequenced the LCMV glycoprotein (GPC), nucleoprotein (NP), and Z protein genes because of their documented participation in viral attachment, fusion, replication, and spreading of infectious virions [30], [31], [32], [33]. Sequencing results of plaque purified clones (3 per animal) revealed no mutations in the Z protein. One viral isolate extracted from the brain of a day 140 mouse had a single amino acid substitution of phenylalanine to leucine (F260L) in the GP1 region of GPC. The F260L mutation has been reported in the immunosuppressive strain of LCMV referred as clone 13 and is thought to give this virus a replicative advantage in peripheral tissues (not the brain) [32], [34]. However, this mutation was not observed in any of the other isolates extracted from the day 140 time point. In addition, no GPC mutations were observed at any other time point. Analysis of the nucleoprotein revealed very similar results. A single amino acid substitution from isoleucine to valine at position 323 (I323V) was detected in one viral isolate at day 15 and two viral isolates at day 140; however, this mutation was sporadic and not likely to influence viral tropism. Thus, our results demonstrate that LCMV was remarkably stable as it established persistence in the brains of OT-I mice and no consistent mutation could explain the observed tropism shift.

### LCMV persistence induces an innate immune gene signature that subsides over time

**Figure 2:**
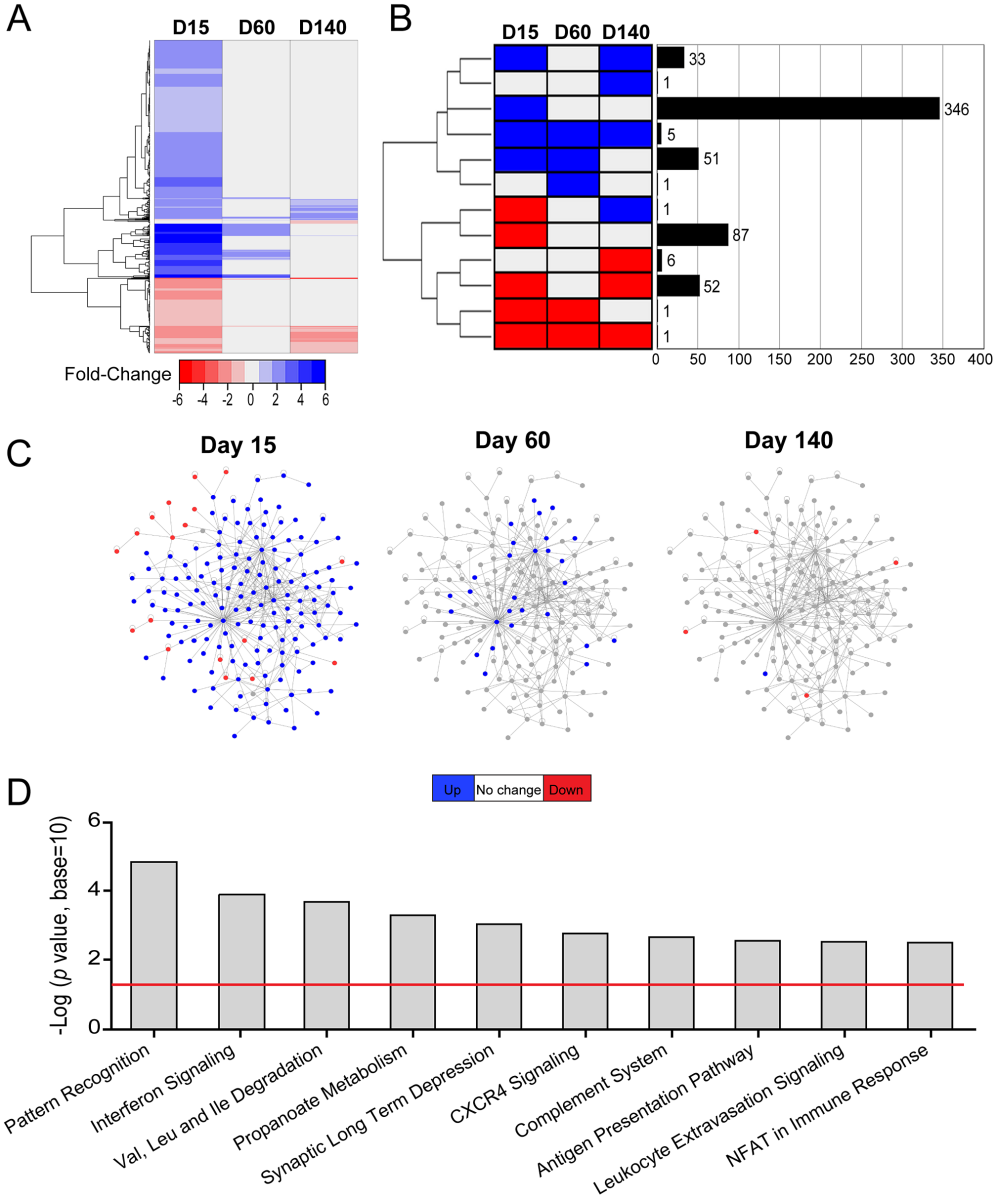
LCMV infection induces a massive change in innate immune gene expression that subsides over time. Microarray analysis was performed on RNA extracted from the brains of LCMV-infected OT-I mice at days 15, 60, and 140 post-infection and compared to mock-infected mice (n = 3 mice per group). (A) A representative heat map shows expression of 596 differentially regulated genes (514 annotated, 82 non-annotated) that were statistically different (*p*<0.05) from mock-infected controls and exceeded a threshold of 1.5-fold. Each column represents the mean fold change of the experimental group relative to the mock-infected group. Genes are clustered together based on functional relatedness (black dendrogram). Blue denotes genes that are up-regulated, red genes are down-regulated, and gray indicates no change. See corresponding Table S1. (B) Genes were clustered and represented graphically based on their expression pattern over the course of infection. Eleven different patterns of gene expression emerged (blue = up-regulation, red = down-regulation, gray = no change). The black bars depict the number of genes that fall into each category. The majority of the genes was either up- (346 genes) or down- (87 genes) at day 15 and returned to baseline thereafter. (C) The protein interaction networks represent 170 genes that were revealed by Ingenuity Pathway Analysis (IPA) software as having direct connectivity with one another. These genes are depicted as nodes on the network, and connecting lines indicate a direct connection. Blue nodes = up-regulated genes, red nodes = down-regulated genes, gray nodes = no change. Note that the network is highly dynamic at day 15 post-infection and then subsides over time. (D) Ingenuity was used to generate a bar graph showing that top 10 biological pathways that were up-regulated in the brains of OT-I mice at day 15 post-infection. The negative log *p* value is plotted on the *y* axis and the red line represents *p*<0.05.

Given our inability to explain LCMV tropism through viral mutation, we next sought insights into the innate immune pressures placed on the virus over time. Infection of OT-I mice provided an ideal opportunity to study pure innate immunity without the confounding influences of an adaptive immune response. Gene array analyses were performed on total brain RNA extracted from LCMV-infected OT-I mice at the denoted time points and compared to a mock-infected control group (Fig. 2, Table S1). Of the 35,556 candidates represented on the microarrays, 585 genes showed a statistically significant (*p*<0.05) difference in expression of at least 1.5-fold (Table S1), and among these 504 had a known gene annotation. As depicted in the heat map (Fig. 2A), most alterations in gene expression (up or down) were observed at day 15 post-infection and then returned to baseline levels as LCMV established widespread persistence in the brain parenchyma at later time points (days 60 and 140). When genes were clustered together based on their pattern of expression (up-regulation, down-regulation, or no change) at the different time points post-infection, it was revealed that most genes were up-regulated at day 15 and returned baseline levels by day 140 post-infection (n = 397 genes) (Fig. 2B, Table S1). A small subset of genes were phasic (n = 33), meaning that they were up at day 15, back to baseline at day 60, and up again at day 140. Most remaining genes were classified into the category of being down-regulated, with the majority being down-regulated at the day 15 time-point (n = 87). Interestingly, several genes remained down-regulated as LCMV shifted tropism during the last stages of viral persistence (n = 59). A complete summary of all differentially regulated genes is provided in Table S1.

To establish the identity and interactions between innate immune genes that responded in the LCMV-infected brain, we used Ingenuity Pathway Analysis (IPA) software to construct a protein interaction network (Fig. 2C) and define the pathways that were most active at the early time point (day 15) post-infection (Fig. 2D). The protein interaction network in Fig. 2C provides a graphical representation of genes identified by IPA as interacting with one another and can be used to infer a coordinated response (see Table S1 for a complete list of genes used to construct this network). Interestingly, at day 15 post-infection, the network was highly dynamic, with most genes being up-regulated (blue). This response was mostly quenched by day 60 and was in fact shifted toward down-regulation (red) by the late phase of infection (day 140). These data suggested that the establishment of LCMV persistence in the brain was associated with a decline in innate immune responsiveness. To gain support for this concept, we next used IPA to identify the top 10 pathways that were active at day 15 post-infection (Fig. 2D, Table S1). As expected, most active pathways at this time were associated with immunological activity (e.g. pattern recognition, interferon signaling, antigen presentation, *etc*); however, a negative impact on nervous system function (e.g. synaptic long term depression) was also observed. In fact, several genes responsible for nervous system function remained depressed at day 140 post-infection (see Table S1 for examples).

**Figure 3:**
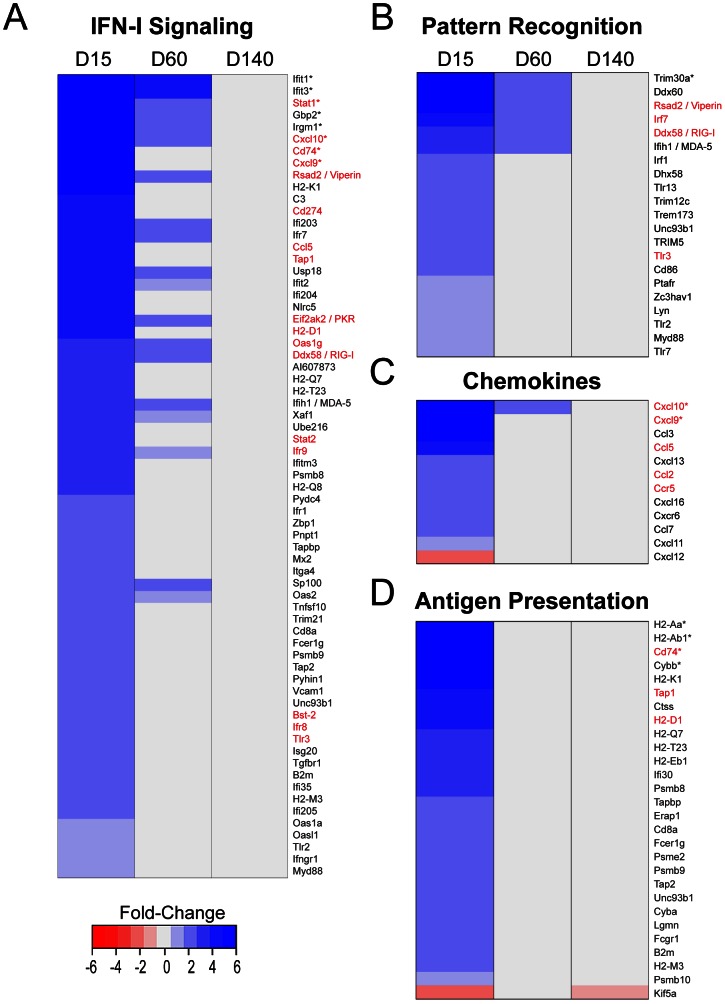
Innate immune gene expression is abrogated during chronic LCMV persistence. Representative heat maps are shown for differentially regulated immunological genes (*p*<0.05, fold expression ≥1.5) associated with IFN-I signaling (A), pattern recognition (B), chemokines (C), and antigen presentation (D) at days 15, 60, and 140 post-infection. Genes associated with the aforementioned immune functions were defined using IPA software. Blue nodes = up-regulated genes, red nodes = down-regulated genes, gray nodes = no change. Asterisks denote genes with a fold-change of greater than 6. Genes written in red text were validated by Q-PCR (see Figures S1 & S2). Note that all genes return mock-infected levels by 140 days post-infection.

Immunologically, the brain was highly dynamic at day 15 post-infection and showed a very marked signature linked to type I interferon (IFN-I) signaling (Fig. 3A). In addition, genes associated with pattern recognition (Fig. 3B), chemoattraction (Fig. 3C), and antigen presentation (Fig. 3D) were also highly active, fitting with the development of a robust innate immune response. To validate the microarray data, we performed quantitative PCR analysis for 20 randomly selected immunological genes (Fig. S1, S2; Table S2). There were no discrepancies between the microarray and Q-PCR data, which confirmed the fidelity of our microarray approach. Overall, the immunological response to LCMV at day 15 was quite robust and included most of the genes that typically set the stage for an efficient anti-viral response. Interestingly, many genes had returned to baseline expression levels by day 60, and the entire innate immune response to LCMV was largely silenced by day 140 (Fig. 3, S1, S2). These data demonstrate that an initially robust response to LCMV subsides as the virus establishes long term persistence in the brain.

### Innate myeloid cell dynamics return to a “naïve” state during viral persistence

**Figure 4:**
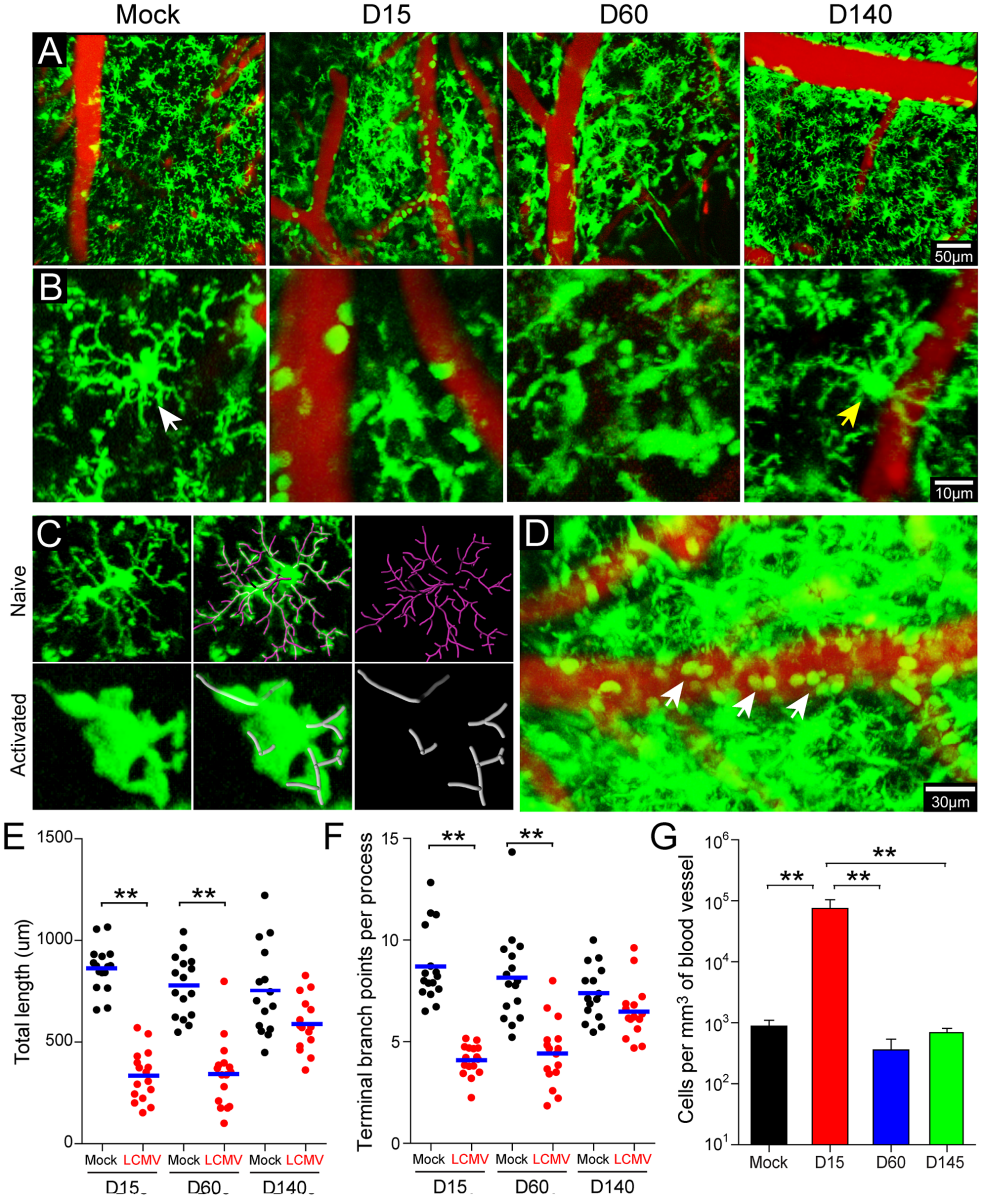
Dynamics of innate myeloid cells in the brain during viral persistence. TPLSM was performed through a surgically thinned skull window in mock-infected OT-I CX3CR1-GFP^+/−^ control mice and compared to LCMV-infected mice at days 15, 60, and 140 post-infection. (A, B) Representative low (A) and high (B) magnification maximal projections of two-photon *z* stacks (50 µm depth) show the distribution of innate myeloid cells (green) in relation to cerebral blood vessels (red). At day 15 post-infection, cerebral blood vessels are more heavily patrolled by GFP^+^ cells and parenchymal myeloid cells (microglia) become less ramified. This response subsides by day 140 post-infection. The white and yellow arrowheads denote ramified microglia in mock-infected and day 140 infected mice, respectively. See corresponding Movie S1. (C, D) Imaris software was used to quantify microglia branch length and complexity (C) as well as the number of GFP^+^ cells per mm^3^ of cerebral blood vessel (D) in mock- and LCMV-infected mice. Panel C shows the branch complexity of representative microglia from mock (upper panels, pink filaments) versus day 15 LCMV-infected mice (lower panels, gray filaments). See corresponding Movie S2. Panel D shows elevated patrolling of cerebral blood vessels by GFP^+^ cells (white arrowheads) at day 15. See corresponding Movie S3. (E, F) Dot plots depict microglia branch length (E) and number of termini (F) in mock vs. LCMV-infection at days 15, 60, and 140 post-infection. Each dot represents a single cell (n = 16 microglia randomly selected from 4 different mice). Asterisks denote statistical significance (*p*<0.05) and blue lines denote the mean of the group. (G) The bar graph shows the number of GFP^+^ cells per mm^3^ of cerebral blood vessel (mean ± SEM; n = 4 mice per group). Asterisks denote statistical significance (*p*<0.05) and data are representative of at least two independent experiments.

The CNS is equipped with an elaborate network of innate immune sentinels that inhabit the meninges and parenchyma [13], [35]. Microglia, the most abundant CNS-resident myeloid cells, possess highly dynamic processes that continually scan the extracellular space in the uninflamed brain [8]. In general, myeloid sentinels are among the first responders to CNS perturbations and thus serve as a barometer for the state of immunological activity in this tissue. Our microarray data established a time line during which innate immune gene expression subsided as LCMV established persistence. To determine how innate myeloid cells responded to LCMV over this time line, we imaged the meninges and underlying brain parenchyma of infected vs. mock-infected OT-I CX3CR1-GFP^+/−^ mice through a surgically thinned skull window [36] using intravital two-photon laser scanning microscopy (TPLSM) [24], [35]. CX3CR1-GFP^+/−^ reporter mice [37] were used because brain-resident (e.g. microglia, meningeal macrophages, *etc.*) as well as circulating (e.g. monocytes) myeloid cells can be visualized [37]. At day 15 post-infection, we observed a marked transformation in the morphology and dynamics of CX3CR1-GFP^+/−^ cells (primarily microglia) in the brain parenchyma (Fig. 4A–B; Movie S1). Relative to mock-infected controls, microglia from day 15 mice had enlarged somas, were amoeboid in shape, and had retracted cell processes. This was quantified by measuring microglia branch length and complexity in TPLSM *z*-stacks (Fig. 4C, E, F; Movie S2). These data revealed a statistically significant reduction (*p*<0.05) in microglia branch length (Fig. 4E) and complexity (Fig. 4F) at day 15 post-infection, which is indicative of activation. In addition, the luminal surface of blood vessels was heavily patrolled by GFP^+^ cells (presumably monocytes) at day 15 (Fig. 4D, Movie S3). Quantification revealed a nearly 2 log increase in the number of GFP^+^ cells scanning the vasculature at this time (Fig. 4G). By day 60 post-infection, the CX3CR1-GFP^+/−^ microglia retained their enlarged, amoeboid morphology (Fig. 4A–C, E–F; Movie S1); however, vascular patrolling had declined to the levels observed in mock-infected control mice (Fig. 4G; Movie S3). Interestingly, at day 140 post-infection, despite extensive viral persistence, myeloid cell activity in the brain had returned to a quasi-normal state (Fig. 4; Movies S1, S3). Although microglia branch length and complexity was slightly reduced, the values were comparable to that observed in mock-infected control mice (Fig. 4E, F). Vascular patrolling had also returned to baseline levels (Fig. 4G). These data indicate that myeloid cell dynamics over time mirrored the pattern of innate immune gene expression following LCMV infection, with both subsiding as the virus established persistence in the parenchyma.

### IFN-I signaling restricts LCMV infection and induces the innate immune program to the virus

**Figure 5:**
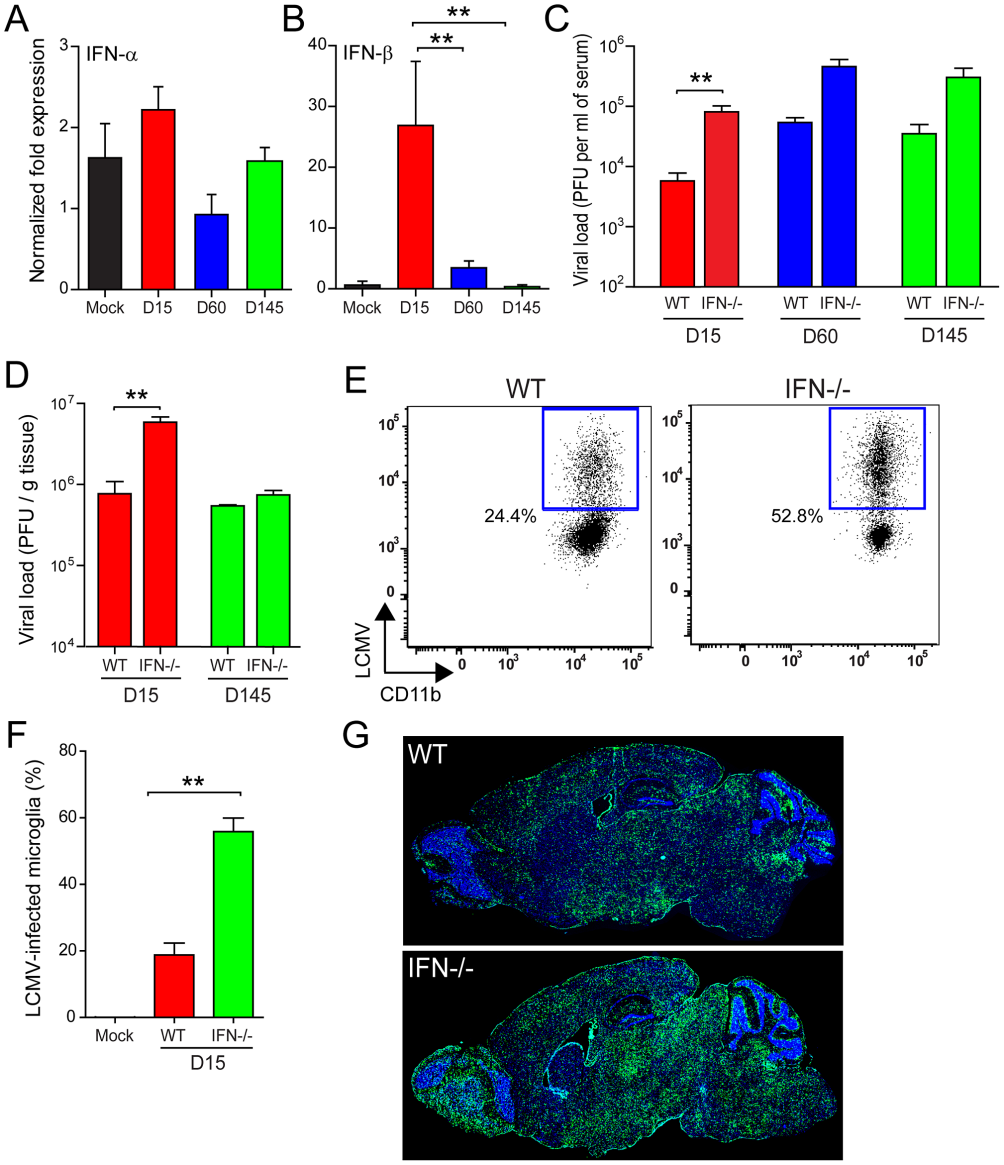
IFN-I is expressed and restricts LCMV infection of the brain during viral persistence. (A, B) Expression of IFN-α (A) and IFN-β (B) mRNA was quantified by Q-PCR at the denoted time points post-infection and compared to mock-infected control mice (n = 3 mice per group). A significant increase in IFN-β expression (asterisks, *p*<0.05) was observed in the brains of OT-I mice at days 15 and 60 post-infection. No significant change in IFN-α was observed using universal primers that detect 13 different IFN-α species. (C, D) Viral load was quantified by plaque assay using sera and brain tissue from OT-I vs. OT-I IFN-IR^−/−^ mice (n = 3 mice per group) at the indicated time points. Asterisks denote statistical significance (*p*<0.05). (E, F) At day 15, infection of brain resident microglia was analyzed flow cytometrically in OT-I vs. OT-I IFN-IR^−/−^ mice (n = 4 mice per group). Representative FACS plots show the percentage of LCMV-infected microglia (gated on CD45^low^ Thy1.2^−^ Gr-1^−^ CD11b^+^ cells). The percentage represents the mean of the group. Bar graphs show the mean ± SEM for the indicated groups and asterisks denote statistical significance (*p*<0.05). (G) Representative sagittal brain reconstructions show the distribution of LCMV (green) in OT-I vs. OT-I IFN-I R^−/−^ mice (n = 4 mice per group) at day 140 post-infection. Nuclei are shown in blue. Note that the distribution of LCMV is greater in OT-I IFN-I R^−/−^ mice relative to the OT-I control group. All data in this figure are representative of at least two independent experiments.

Because our microarray data revealed a gene expression signature that was linked in part to IFN-I signaling, we next quantified the expression of IFN-α (13 subsets using universal primers) [38] and IFN-β1 in the brain by Q-PCR (Fig. 5A, B). At day 15 post-infection, a significant (*p*<0.05) 26-fold increase in IFN-β1 expression was detected in the brain and IFN-α expression trended upward, although it did not reach statistical significance. IFN-β1 levels dramatically declined by day 60 post-infection and returned to baseline by day 145 post-infection. To evaluate whether IFN-I exerted anti-viral pressure on LCMV during the early or late stages of viral persistence, we quantified viral loads in OT-I mice lacking the IFN-I receptor (OT-I IFNR^−/−^). Increased viral loads were observed in the serum of OT-I IFNR^−/−^ at all time points post-infection, demonstrating that peripheral viral loads are influenced by IFN-I (Fig. 5C). Interestingly, viral titers were significantly increased (*p*<0.05) in the brains of OT-I IFNR^−/−^ only at day 15 post-infection (Fig. 5D). No difference was noted at day 145, which was consistent with the reduction of IFN-I to baseline levels by this time point (Fig. 5A, B). These data suggested that IFN-I exerted anti-viral pressure only during the early stages of LCMV persistence in the brain. To directly test this assertion, we measured the degree to which LCMV penetrated the brain parenchyma in OT-I vs. OT-I IFNR^−/−^ at day 15 post-infection. This was accomplished by flow cytometrically quantifying the percentage of microglia (a representative parenchyma cell) that contained LCMV antigen. In the absence of IFNR, a marked increase in the percentage of LCMV^+^ microglia was detected (Fig. 5E, F), suggesting that the virus was able to invade the parenchyma more efficiently. This ultimately resulted in a greater abundance of viral antigen in the brain parenchyma during the late stage (day 140) of persistence (Fig. 5G).

**Figure 6:**
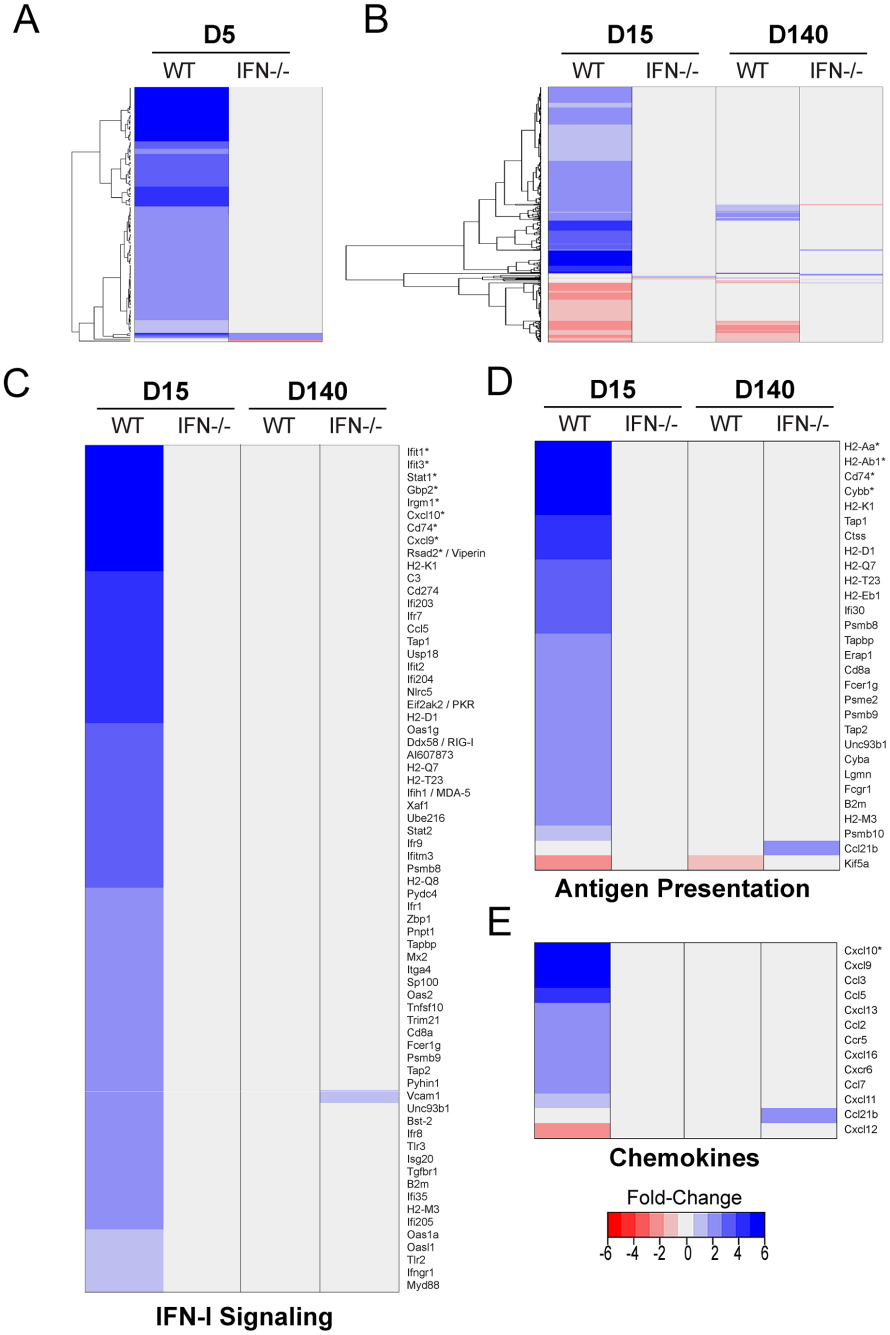
IFN-I receptor deficiency abrogates nearly all gene expression in the LCMV-infected brain. Microarray analysis was performed on RNA extracted from the brains of LCMV-infected OT-I vs. OT-I IFN-I R^−/−^ mice at days 5, 15 and 140 post-infection. Mock-infected mice from both strains were used as controls for this experiment (n = 3 mice per group). (A) A representative heat map shows expression of 141 differentially regulated genes (137 annotated, 3 non-annotated) that were statistically different (*p*<0.05) from mock-infected controls at day 5 post-infection and exceeded a threshold of 1.5-fold. Each column represents the mean fold change of the experimental group relative to the mock-infected group. Genes are clustered together based on functional relatedness (black dendrogram). Blue denotes genes that are up-regulated, red genes are down-regulated, and gray indicates no change. See corresponding Table S1. (B) A representative heat map shows expression of 585 differentially regulated genes (504 annotated, 81 non-annotated) that were statistically different (*p*<0.05) from mock-infected controls at days 15 and 140 post-infection and exceeded a threshold of 1.5-fold. Note that nearly all genes remain unchanged in LCMV-infected OT-I IFN-IR^−/−^ mice. See corresponding Table S1. (C–E) Analysis of selected individual pathways (IFN-I signaling, antigen presentation, and chemokines) using AmiGO and IPA revealed no change in OT-I IFN-IR^−/−^ mice at days 15 or 140 post-infection except an increase in VCAM1 and CCL21b expression at day 140. Asterisks denote genes with a fold-change of greater than 6.

The IFN-I expression pattern and its anti-viral activity in the brain supported a role for this innate cytokine in the defense against LCMV. However, the impact of IFN-I on the global anti-viral program remained unclear. To address this question, we used a microarray approach to quantify the genes that were differentially regulated in the brains of LCMV-infected OT-I vs. OT-I IFNR^−/−^ at early and late stages of persistence (Fig. 6, Table S1). Surprisingly, deletion of IFN-I signaling resulted in near complete inactivation of the entire anti-viral program at days 5, 15, and 140 post-infection. At day 5 post-infection, 137 annotated genes were differentially expressed in OT-I mice relative to the mock-infected control group. Among these only three genes were upregulated in IFNR^−/−^ mice: LCN2 (2.46-fold), H2-K1 (1.73-fold), and GBP4 (1.66-fold). The number of annotated, differentially expressed genes increased to >500 by day 15 post-infection in OT-I mice; however, only 1 gene (HSPA1b) was upregulated in IFNR^−/−^ mice, which is not associated with immune function. In fact, focused heat maps revealed that no innate immune genes were up- or down-regulated in IFNR^−/−^ mice at day 15 or 140 post-infection besides VCAM1 and CCL21b (Fig. 6C). Further analyses at day 140 post-infection uncovered only 3 additional upregulated genes (SCRG1, LY86, SFT2D2) in IFNR^−/−^ mice. In essence, LCMV became invisible in the brain of its murine host. These data indicate that almost all differential gene expression observed in LCMV-infected brains is either directly or indirectly linked to IFN-I signaling.

### IFN-I signaling triggers all innate myeloid cell dynamics in the LCMV-infected brain

**Figure 7:**
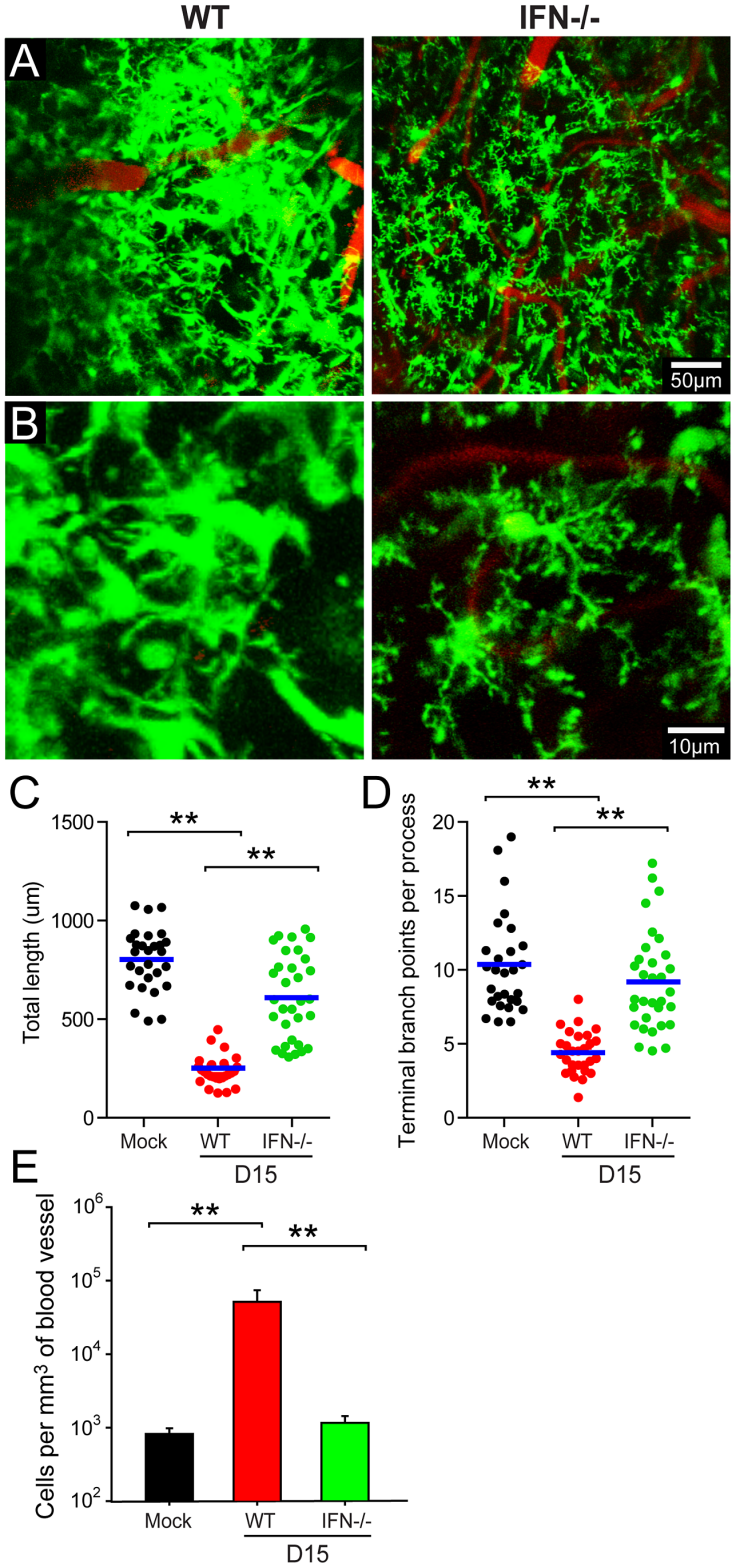
IFN-I regulates the activation and dynamics of all innate myeloid cells in the persistently infected brain. TPLSM was performed through a thinned skull window of OT-I CX3CR1-GFP^+/−^ and OT-I IFN-IR^−/−^ CX3CR1-GFP^+/−^ mice at day 15 post-infection (n = 4–8 mice per group). Mock-infected mice were used as controls for this experiment. (A, B) Representative low (A) and high (B) magnification maximal projections of two-photon *z* stacks (50 µm depth) show the distribution of innate myeloid cells (green) in relation to cerebral blood vessels (red). Note that parenchymal myeloid cells (mostly microglia) remain non-activated and highly ramified in LCMV-infected OT-I IFN-IR^−/−^ CX3CR1-GFP^+/−^ mice relative to wild type controls. See corresponding Movie S4. (C, D) Quantification of microglial branch length (C) and complexity (D) was performed for the denoted groups of mice. Each dot represents an individual, randomly selected microglia. Blue bars represent the mean of the group. (E) The bar graph shows the number of GFP^+^ cells per mm^3^ of cerebral blood vessel (mean ± SEM; n = 4 mice per group). Asterisks denote statistical significance (*p*<0.05). Data are representative of at least two independent experiments.

Given that IFN-I induced all gene expression in the LCMV-infected brain, we set out to determine whether this pathway was responsible for innate myeloid cell dynamics. This is a particularly important question because innate immune cells like microglia can respond within minutes to soluble cues (e.g. ATP mediated purinergic receptor signaling) [9] that do not necessarily require changes in gene expression. To address how CNS myeloid cells respond to LCMV in the absence of IFN-I signaling, we imaged the brains of OT-I IFNR^−/−^ CX3CRI-GFP^+/−^ mice by TPLSM at day 15 post-infection – a time point when a robust innate myeloid response is typically observed (Fig. 4). Interestingly, our TPLSM studies revealed that IFNR^−/−^ mice were completely unable to sense and respond dynamically to LCMV (Fig. 7; Movie S4). In the absence of IFN-I signaling, microglia remained ramified and their branch length and complexity was comparable to that observed in mock-infected control mice (Fig. 7A–D). This was particularly interesting given that the percentage of LCMV-infected microglia increased 2-fold in IFNR^−/−^ mice (Fig. 5E, F); thus, there was a greater potential for these cells to be directly stimulated by LCMV. As another measure of innate myeloid activity, we quantified the amount of vascular patrolling by CX3CRI-GFP^+/−^ cells in the presence or absence of IFNR^−/−^ (Fig. 7E). Similar to the microglia data, vascular patrolling in IFNR^−/−^ was comparable to that observed in mock-infected controls. In concert, these results indicate that IFN-I signaling not only induces gene expression but is also responsible for innate myeloid cell dynamics following LCMV infection of the brain.

## Discussion

Innate protection of the CNS is mediated by an elaborate network of innate immune sentinels that consist of microglia, specialized macrophages, and DCs [13]. These often serve as the first responders against invading infectious agents and must hold these microbes in check prior to the arrival of adaptive immune cells. In this study, we unexpectedly uncovered that the brain has an Achilles' heel in its defense against a non-cytopathic arenavirus. Specifically, all gene expression and innate myeloid cell dynamics were completely abrogated in the absence of IFN-I signaling. That LCMV induced an IFN-I signature of gene expression in the brain was not surprising given that the virus is detected by RIG-I/MDA5 and is known to trigger IFN-I production [22]. It was not predicted, however, that all differentially regulated genes (both up and down) would be linked exclusively to this pathway. During the early stage of infection, when LCMV localized primarily to the meninges and superficial parenchyma, a robust innate response was observed at the genomic and cellular levels. At this time point, 5 logs of infectious virus were detected in the brain, 585 genes were differentially regulated, microglia had transformed morphologically, and vascular patrolling by innate myeloid cells was markedly elevated. These were all indicators of a successful innate response. During the later stages of persistence, no consistent pattern of viral mutation was observed, nor was there any gain in the efficiency of infectious virion generation. However, both IFN-β synthesis and the network of innate immune gene expression were largely silenced as the virus moved into the brain parenchyma. This coincided with the restoration of myeloid cell dynamics to a quasi-normal state by 140 days post-infection, suggesting equilibration between LCMV and its murine host. Importantly, when IFNR^−/−^ mice were infected with LCMV, no anti-viral response was observed at the genomic or dynamic levels despite increased viral loads and infection of microglia. These data indicate that the brain has only one way to respond innately to LCMV. This Achilles' heel has in turn been exploited by LCMV and most other arenaviruses, which were demonstrated *in vitro* to suppress IFN-I synthesis and signaling [22], [39], [40], [41]. This could also explain why many other neurotropic viruses have acquired strategies to dampen the IFN-I pathway [20].

Because LCMV showed no discernable mutation pattern as it invaded the brain, we focused instead on the evolution of the innate inflammatory response, which is known to influence the tropism and virulence of many neurotropic viruses [19]. During the early stages of persistence, LCMV elicited a classic innate response that appeared capable of supporting a successful adaptive response (which never followed due to the restricted T cell repertoire in OT-I mice). The innate program was coordinated and highly interactive. In fact, 170 gene products were assembled into a protein interaction network identified using IPA software (see Table S1 for individual genes). Genes associated with pattern recognition (e.g. *TLR2, TLR3, TLR7, MyD88, RIG-I, PKR, MDA-5*), antigen presentation (e.g. *MHC I, MHC II, B2M, TAP1, TAP2*), chemoattraction (e.g. *CCL2, 3, 5, 7, 9, 10*), and IFN-I signaling (e.g. *IRF7, IRF8, IRF9, STAT1, STAT2*) were all highly expressed at this time point (refer to Table S1 for a complete list). Innate anti-viral genes were also highly expressed at day 15. Some of the best described anti-viral factors included dsRNA activated protein kinase (*PKR*), 2′-5′ oligoadenylate synthatases (*OAS1g, OAS1b, OAS2 and OASL*), and 3′ to 5′ exonuclease specific to ssRNA (*ISG-20*). We also observed increased expression of several other anti-virals, which include but are not limited to bone marrow stromal cell antigen 2 (*BST-2*), viperin, interferon-induced transmembrane protein 3 (*IFITM3*), interferon-induced protein with tetratricopeptide repeats 1 (*IFIT1*), guanylate binding protein 2 (*GBP-2*), and the anti-viral helicase, DDX-60. Studies have shown that these gene products directly interfere with the replication of several mammalian viruses, including human immunodeficiency virus (*BST-2*) [42], influenza A (viperin) [43], Influenza A/West Nile/Dengue virus (*IFITM3*) [44], [45], vesicular stomatitis virus/encephalomyocarditis virus (*GBP2*) [46], and hepatitis C virus (*DDX-60*) [17]. However, the importance of these factors in restricting the replication of LCMV in the brain remains unknown. Recent studies have demonstrated that IFN-I induces expression of many different anti-viral factors, some broadly acting and others with targeted specificity [17]. Given the breadth of factors uncovered at day 15, it is likely that IFN-I expression induces a generic anti-viral state in the brain capable of handling a variety of different infections.

As LCMV moved into the deeper brain parenchyma, a dramatic reduction in IFN-β production and overall innate immune gene expression was observed. During this transition, the number of viral genome copies increased, but the amount of infectious virus remained constant, suggesting that defective interfering particles were generated [47], [48]. A previous study demonstrated that LCMV downregulates GP expression during persistence in the CNS [49], which could explain the stable set point of infectious virus observed in the brains of OT-I mice over time. Even more interesting, however, was the steady decline in innate immune gene expression that proceeded to a near complete silencing by the late stage of persistence (day 140). There are two conceivable explanations for this result. First, there are many negative regulators of IFN-I production [50], and we found that two were increased in expression at days 5 and 15 post-infection (*NLRC5* and *TRIM21*). NLRC5 is a NOD-like family protein recently shown to block phosphorylation of IκB kinase (IKK) complex, which in turn reduces nuclear factor κB (NF-κB) activation [51]. NLRC5 also interacts with RIG-I/MDA5 and impedes their function. Importantly, siRNA mediated silencing of NLRC5 resulted in increased IFN-I production by VSV-infected cells [51]. The other negative regulator detected in the brains of OT-I mice was the E3 ligase Ro52 (TRIM21). This protein was shown to interact with IRF3 and promote its degradation, thus decreasing IFN-β promoter activity [52]. The upregulation of both NLRC5 and TRIM21 at days 5 and 15 could explain the decline in IFN-I expression by day 60. Because chronic IFN-I production has the potential to disrupt neurological function, negative regulation could be a strategy used by the brain to quench the innate response.

Another explanation for the decline in CNS immune activity is that LCMV and most other arenaviruses tested can dampen IFN-I production [22], [39], [40], [41]. This activity maps to the C-terminal region of the LCMV NP [39], [40], which binds to the kinase domain of IKKε preventing it from phosphorylating IRF3 [41]. This consequently reduces the production of IFN-β. Given that most arenaviruses have acquired a similar strategy to quench IFN-I production, it is likely that this property provides a replicative advantage *in vivo* and perhaps an improved ability to equilibrate with their host during persistence. Of particular interest in the brain is the fact that the entire gene expression profile following LCMV infection is linked to IFN-I signaling. We detected no differentially regulated genes in IFNR^−/−^ mice, and the virus was able to more efficiently invade the brain parenchyma. Thus, it seems reasonable to conclude that LCMV has evolved a strategy to mirror an IFN-I receptor knockout mouse, because this is the only innate pathway available to the brain following infection. This theory is further supported by studies showing that the IFN-I signature is also minimal in mice persistently infected from birth with LCMV (referred to as carrier mice) [53], [54]. Therefore, suppression of IFN-I appears to be a cardinal feature of LCMV persistence.

At the dynamic level, the innate myeloid response to LCMV was also quite fascinating. Recent TPLSM studies have demonstrated that brain resident myeloid cells like microglia are remarkably dynamic (even in the naïve brain) and can rapidly respond to inflammatory challenges [8], [9]. To date, investigators have focused primarily on microglial responses to parenchymal injuries. Following laser injury, for example, microglia were shown to extend processes within minutes toward the site of damage, and this response was dependent on purinergic receptor signaling [8], [9]. At the outset of our studies, it was unclear whether brain myeloid cells would respond similarly to a viral infection. Interestingly, we observed that microglia responded innately to a LCMV infection by transforming into an amoeboid morphology and reducing branch length/complexity. This program is likely set into motion to sequester virus and facilitate antigen presentation, which is supported by our microarray data showing that many anti-viral and antigen presentation genes are up-regulated at day 15. We also observed a marked increase in vascular patrolling by myeloid cells at this time point, fitting with increased expression of the adhesion molecule, VCAM1, as well as production of myelomonocytic recruiting chemoattractants like CCL2, 3, and 5. CX3CR1-GFP^+/−^ monocytes were shown previously to patrol the luminal surface of blood vessels [55], and these cells are known to enter the brains of LCMV-infected mice [24]. Based on our imaging, genomic, and knockout data, IFN-I initiates the program that facilitates the arrival of innate myeloid cells. Over time, however, myeloid cell dynamics subside in the LCMV-infected brain, eventually returning to a quasi-normal state despite widespread viral persistence. This coincided with a decline in IFN-I production, which suggested that IFN-I (rather than purinergic) signaling was responsible for all innate myeloid cells dynamics. We proved this definitively by studying immune cell dynamics in IFNR^−/−^ mice. In the absence of IFN-I signaling, innate myeloid cells in the LCMV-infected brain behaved similarly to mock-infected controls. This result demonstrated that IFN-I alone orchestrated all innate immune gene expression and myeloid cell dynamics following LCMV infection, which is surprisingly rare example of a tissue having no redundant mechanisms in place to mount a response.

We believe that the implications of findings are broad and may facilitate the design of therapeutics to increase or decrease anti-viral immunity in the CNS. The importance of IFN-I signaling in the LCMV model was clearly demonstrated by Muller and colleagues who showed that IFNR^−/−^ mice do not develop LCMV induced meningitis, but instead become asymptomatic viral carriers [56]. Based on our findings, this result is likely explained by a near complete shutdown of all innate immune activity in the brains of IFNR^−/−^ mice. Recent studies have also shown using a murine model of cerebral malaria induced by the parasite, plasmodium *berghei*, that IFNR^−/−^ mice do not develop fatal disease [57]. AT-rich motifs in the plasmodium genome trigger IFN-I production, which appears to facilitate the development of cerebral malaria. Thus, it will be important to identify the innate immune signature induced in the brains of plasmodium infected mice in the presence or absence of IFN-I signaling. It will also be important to determine how much the brain relies on IFN-I for its response to other neurotropic viruses. A recent study demonstrated that IFN-I production by innate myeloid cells in the lymphatics can protect peripheral nerves from a fatal VSV infection [58], but it remains to be determined whether IFN-I is the exclusive pathway used to innately protect the nervous system. Our prediction is that cytopathic neurotropic viruses will trigger other innate pathways in the brain due to the release of DAMPs associated with direct cellular injury. Detection of non-cytopathic viruses, on the other hand, may be more dependent on IFN-I release. Arenaviruses like LCMV appear to exploit this fact to become invisible in their hosts. We too may be able to exploit the brains exclusive reliance on IFN-I to develop therapies that modulate immunity to CNS infections.

## Methods

### Transgenic mice

C57BL/6 (B6), B6-Tg(TcraTcrb)1100Mjb/J (OT-I) [27], [28], and B6 CX3CR1-GFP^+/+^ [37] mice were purchased from The Jackson Laboratory. OT-I and CX3CR1-GFP^+/+^ mice were then maintained in a closed breeding facility at The National Institutes of Health (NIH). B6 IFN-I receptor^−/−^ (IFN-IR^−/−^) [56] mice were generously provided by Dr. Jonathan Sprent (formerly at The Scripps Research Institute). OT-I IFN-IR^−/−^, OT-I CX3CR1-GFP^+/−^ and OT-I IFN-IR^−/−^ CX3CR1-GFP^+/−^ mice were generated by interbreeding the aforementioned strains. This study was carried out in strict accordance with the recommendations in the Guide for the Care and Use of Laboratory Animals of the National Institutes of Health. The protocol was approved by the NINDS Animal Care and Use Committee (Assurance Number: 1295-12). All experimental procedures were performed under anesthesia and conducted in a manner minimize suffering.

### Virus

Animals 6–8 weeks old were intracerebrally (i.c.) infected with 10^3^ PFU LCMV Armstrong (Arm). Stocks were prepared by single passage in BHK-21 cells at a low multiplicity of infection (MOI of 0.01), and the titer was determined by plaque assay in Vero cells.

### LCMV genome screening

Brain tissue extracted from mice that received an intracardiac perfusion with 25 ml of saline were homogenized in 1 ml of RPMI and centrifuged at 16,000 g for 5 min. The supernatant fraction was used to determine the viral titer by plaque assay on Vero cells. Subsequently, plaque purified viral isolates were selected for RNA extraction using a PureLink Viral RNA/DNA mini kit (Invitrogen). The RNA was then reverse transcribed and PCR amplified by using gene specific primers. The following primers were used for PCR amplification and sequencing: GPC-Fwd (ATGGGTCAGATTGTGACAATG), GPC-660Fwd (CATGATTTACATTGCATTTC), GPC-Rev (TCAGCGTCTTTTCCAGACGG), Z-Fwd (GCACCGGGGATCCTAGGC), Z-Rev (GTGTGTGTGTGTGGGTGTGCGTG), NP-Fwd (ATGTCCTTGTCTAAGGAAG), NP526-Fwd (CAATCAATTTGGCACAATGC), NP1215-Fwd (CGTTGATCAAAAACAATTCAAG), NP-Rev (TTAGAGTGTCACAACATTTGGG).

### Real time PCR

The pellet fraction from the homogenized brain was treated with Trizol (Invitrogen) and total RNA was extracted by using a PureLink RNA mini kit (Invitrogen). One µg of RNA was then treated with amplification grade DNAse I (Invitrogen) and reverse transcribed with iScript (Bio-Rad) reverse-transcription reagent kit, which contains a mixture of oligo(dT) primers and random hexamers without any gene specific primers. All the Q-PCR reactions were performed in 20 µl volumes using SoFast EvaGreen super mix (Bio-Rad) in a 96-well optic tray on a CFX96 Real-Time PCR machine (Bio-Rad). The reactions were conducted in triplicate, and samples without reverse transcriptase were used as a non template control. The Q-PCR cycling conditions were as follows: initial denaturation at 95°C for 3 min followed by 40 cycles with denaturation at 95°C for 10 seconds, annealing for 10 seconds and extension at 72°C for 20 seconds. The optimal annealing temperature for gene specific primers (see Table S2) was determined prior to the experimental runs. The housekeeping gene beta-actin was used as a reference. For determination of viral genome copy number, Q-PCR for the LCMV glycoprotein was conducted using 10 ng of cDNA and referenced to known quantities of linearized LCMV S-segment plasmid DNA as described previously [29].

### Immunohistochemistry

To visualize LCMV antigen, 6-µm frozen sagittal brain sections were cut with a cryomicrotome and fixed for 10 min with 4% paraformaldehyde in PBS. Sections were then treated with an avidin-biotin blocking kit (Vector Laboratories) per the manufacturer's instructions and incubated overnight at 4°C with a rat anti-LCMV nucleoprotein monoclonal antibody (1∶1000 dilution; VL-4 clone, BioXcell). Following the incubation with primary antibody, sections were washed, incubated with a biotinylated anti-rat secondary (1∶400; 1 hr room temperature; Jackson ImmunoResearch), washed, and incubated with streptavidin-Rhodamine Red X (1∶400; 1 hr room temperature; Jackson ImmunoResearch). Lastly, all sections were stained with 1 µg/ml 4′,6′-diamidino-2-phenylindole (DAPI; Sigma-Aldrich) to visualize cell nuclei. All working stocks of primary and secondary reagents were diluted in PBS containing 2% FBS.

### Epifluorescence microscopy

Two-color reconstructions of sagittal brain sections (Fig. 1A and 5G) were generated using a Zeiss Z1 inverted epifluorescence microscope fitted with an automated *xyz* stage, an Axiocam digital camera, and a 5× objective (Carl Zeiss Microscopy). Individual images were assembled into a composite image using a mosaic feature in the Axiovision acquisition software (Version 4.8, Carl Zeiss Microscopy).

### Intravital two-photon microscopy

For intravital imaging experiments, mice were anesthetized and maintained at a core temperature of 37°C. To generate a viewing window into the brain, skull bones were thinned as described previously [36], [59]. Prior to imaging, mice were injected intravenously with 100 µl normal saline containing Qtracker non targeted quantum dots (655 nm; 0.2 µm; Invitrogen). All 4D imaging data were collected using a Leica SP5 two-photon microscope (Leica) equipped with an 8,000-Hz resonant scanner, a 20×/1.0 NA water-dipping objective, and a Mai Tai HP DeepSee Laser (Spectra-Physics) tuned to 920 nm. Fluorescence emission was separated by high-efficiency custom dichroic mirrors (Semrock) and collected with an NDD4 external detector (Leica). Static 3D images were captured using a *z* step size of 1 µm to a depth of 100 µm beneath the skull bone. 3D time lapses were captured at the same depth using a 2.5 µm *z* step size and a 1 min time interval. All quantitative analyses of two-photon data were performed using Imaris 7.3 software (Bitplane). Microglia were identified as branched GFP^+^ cells residing in the brain parenchyma of CX3CR1-GFP^+/−^ reporter mice and selected randomly by first using the “spots” function in Imaris to identify all cell bodies in a given volume. The identification numbers of each cell were then entered into a randomizer after which individual microglia were sequentially selected from the randomized list for quantification. Only microglia whose processes were all within the field of view were quantified. Microglial branch length and the number of termini were quantified using the “FilamentTracer” feature in Imaris. Branch endpoints, bifurcations, and initiation points were identified on each cell manually and connected using the “FilamentTracer” tool (see Fig. 4C for examples). The resultant tracings were then used to calculate the total process length (µm) and number of terminal branch points per process. A process was defined as an extension originating from the cell body of a microglia. To calculate the number of CX3CR1-GFP^+/−^ cells per unit volume of cerebral vasculature, the “surfaces” tool in Imaris was used to generate volume renderings of blood vessels visualized by intravenously injected 655 nm quantum dots. After calculating vascular volumes (mm^3^), the CX3CR1-GFP^+/−^ cells were counted manually in individual vessels at three time points (0 min, 30 min, and 60 min). This number was then averaged and divided by the vascular volume to obtain cells per mm^3^ of blood vessel.

### Mononuclear cell isolation

Single cell suspensions of brain and spleen were performed after intracardiac perfusion of anesthetized mice with 25 ml of normal saline to remove the contaminating blood lymphocytes. To isolate brain infiltrating leukocytes, minced brains were incubated with 1 ml of collagenase D (1 mg/ml; Roche) at 37°C for 30 min followed by mechanical disruption through a 100 µm filter. Homogenates were then resuspended in 4 ml of 90% Percoll (GE Healthcare) in HBSS, and a Percoll gradient was prepared by overlaying 3 ml of 60% Percoll, 4 ml of 40% Percoll, and 3 ml of 1× HBSS, respectively. The gradients were then centrifuged at 1700 rpm for 20 min at 4°C, after which the band (interface between 60% and 40% Percoll) corresponding to mononuclear cells was isolated, and a single cell suspension was prepared by washing these cells with 1× HBSS followed by RPMI. Splenocytes were prepared by mechanical disruption through a 100 µm filter, which were then treated with red blood cell lysis buffer (ammonium chloride; 0.02 M Tris-HCl and 0.14 M NH_4_Cl; pH 7.2) and washed twice before staining. For all tissues, the absolute number of mononuclear cells was determined prior to flow cytometric analysis.

### Flow cytometry

Mononuclear cells isolated from different organs were blocked with 3.3 µg/ml anti-mouse CD16/CD32 (Fc block; BD Biosciences, clone 2.4G2) in PBS containing 1% FBS for 20 min prior to antibody staining. Single cell suspensions were stained with the following antibodies: anti-CD45.2-FITC (BD Bioscience, clone 104), CD8-Pacific Blue (Invitrogen, clone 5H10), CD4-Qdot 605 (Invitrogen, clone RM4-5 ), CD11b-PE-Cy7 (eBioscience, clone M1/70), Gr1-APC (BD Bioscience, clone RB6-8C5), CD11c-APC-Cy7 (Biolegend, clone N418), Thy1.2-Alexa Fluor 700 (Biolegend, clone 30-H12), NK1.1-PerCP Cy5.5 (BD Bioscience, clone PK136), and CD45.2-APC-Cy7 (BD Bioscience, clone 104). For detection of intracellular LCMV, cells were fixed with 4% PFA, permeabilized with 0.1% saponin, and stained with a rat anti-LCMV nucleoprotein monoclonal antibody (VL-4 clone, BioXcell) directly conjugated to Alexa Fluor 647 using an antibody labeling kit (Invitrogen). Cells were acquired using a digital flow cytometer (Digital LSR II; BD) and flow cytometric data were analyzed with FlowJo software (Version 9.0, Tree Star, Inc).

### Microarray hybridization

Samples were prepared according to Affymetrix protocols (Affymetrix, Inc). RNA quality and quantity was ensured using the Bioanalyzer (Agilent, Inc.) and NanoDrop (Thermo Scientific, Inc.) respectively. For RNA labeling, 200 nanograms of total RNA was used in conjunction with the Affymetrix recommended protocol for the GeneChip 1.0 ST chips. The hybridization cocktail containing the fragmented and labeled cDNAs was hybridized to the Affymetrix Mouse Genome ST 1.0 GeneChips. The chips were washed and stained by the Affymetrix Fluidics Station using the standard format and protocols as described by Affymetrix. The probe arrays were stained with streptavidin phycoerythrin solution (Molecular Probes, Carlsbad, CA) and enhanced by using an antibody solution containing 0.5 mg/mL of biotinylated anti-streptavidin (Vector Laboratories, Burlingame, CA). An Affymetrix Gene Chip Scanner 3000 was used to scan the probe arrays. Gene expression intensities were calculated using Affymetrix AGCC software.

### Microarray data analysis

Gene fragment data summarization and normalization was accomplished using the Expression Console with the “RMA Sketch” option selected (Affymetrix, Inc). Quality was assured via Tukey box plot, PCA scatter plot and correlation-based heat map using functions in “R” (www.cran.r-project.org). Locally Weighted Scatterplot Smoothing (Lowess) modeling of the data (coefficient of variation modeled by mean expression) was used to characterize noise for the system and discard noise-biased data. Differential expression was tested for via ANOVA under BH correction conditions followed by a TukeyHSD post-hoc test. Gene fragments found to have a corrected *p*-value<0.05 by ANOVA and a post-hoc *p*-value<0.05 were deemed to have significant differential expression between the corresponding cell types if the absolute difference of means was ≥1.5-fold. Gene annotations were assigned where possible using MGI (www.informatics.jax.org) and IPA (www.ingenuity.com). IPA was also used to generate a protein interaction network comparing differential expression in LCMV- versus mock-infected mice at days 15, 60, and 140 post-infection (Fig. 2C). Biological function associations were assigned using AmiGO (amigo.geneontology.org) and the differential expression for select functions depicted using the enhanced heat map function in “R” (heatmap.2). Genes downstream of IFN-I signaling were assigned using a combination of gene lists obtained from AmiGO and IPA.

### Statistical analysis

Statistical significance (*p*<0.05) was determined in SigmaPlot 11.0 using a one-way ANOVA for normally distributed data or an ANOVA on ranks for populations with non-Gaussian distributions. Graphs were generated using SigmaPlot 11.0 and GraphPad Prism 5.04.

## Supporting Information

Figure S1 **Validation of innate immune gene expression during viral persistence.** Selected innate immune genes identified by microarray analysis (see Fig. 2 and Table S1) were quantified by Q-PCR using mRNA extracted from the brains of OT-I mice at days 15, 60, and 145 post-infection (n = 3 mice per group). Mock-infected mice were used as a control group. For all Q-PCR reactions, β-actin was used a reference gene to calculate normalized fold-expression. Data are plotted as mean ± SEM. Asterisks denote statistical significance (*p*<0.05).
(TIF)

Figure S2 **Validation of immune gene expression.** Selected innate immune genes identified by microarray analysis (see Fig. 2 and Table S1) were quantified by Q-PCR using mRNA extracted from the brains of OT-I mice at days 15, 60, and 145 post-infection (n = 3 mice per group). β-actin was used a reference gene to calculate normalized fold-expression. Data are plotted as mean ± SEM, and asterisks denote statistical significance (*p*<0.05).
(TIF)

Movie S1 **Temporal changes in innate myeloid cell dynamics during establishment of a persistent viral infection.** Representative time lapses of 3D reconstructions show the dynamics of CX3CR1-GFP^+/−^ labeled myeloid cells (green) captured by TPLSM through a thinned skull of mock or LCMV-infected OT-I CX3CR1-GFP^+/−^ mice at the denoted time points. The maximal projections represent z-stacks (60 µm in depth) imaged over a 56 min time period. The blood vessels are labeled with quantum dots (red). Note the ramified microglia in mock-infected mice become highly activated by day 15. This is evidenced by their enlarged cell bodies and shortened processes. In addition, blood vessels are heavily patrolled by GFP^+^ cells at day 15. This response subsides over time.
(MOV)

Movie S2 **Methodology to quantify microglial morphology.** The “FilamentTracer” feature in Imaris was used to quantify microglial process length and complexity at different time points post-infection. **Part 1**: A representative 3D projection captured in the brain of a naïve OT-I CX3CR1-GFP^+/−^ mouse shows normal microglial morphology prior to infection. Note the small cell body and complex branch structure. The filaments identified using Imaris are overlaid in pink. **Part 2**. For comparison, two activated microglial captured in the brain of a day 15 OT-I CX3CR1-GFP^+/−^ mouse have enlarged cell bodies and shorter, less complex processes. Filament tracings are overlaid in orange (cell#1) and pink (cell#2).
(MOV)

Movie S3 **Kinetics of innate vascular patrolling during the establishment of viral persistence.** Representative time lapses of 3D reconstructions show the dynamics of CX3CR1-GFP^+/−^ labeled myeloid cells (green) patrolling parenchymal blood vessels of mock vs. LCMV-Arm infected OT-I CX3CR1-GFP^+/−^ mice at the denoted time points. The maximal projections represent 50 µm z-stacks imaged over a 51 min time period. Blood vessels were visualized using quantum dots (red). Note that GFP^+^ myeloid cells heavily patrol the luminal of surface of brain vasculature at day 15 post-infection when compared to mock-infected controls. This response subsides at later time points post-infection.
(MOV)

Movie S4 **IFN-I signaling deficiency completely abolishes microglial activation and dynamics following infection.** Representative time lapses of 3D reconstructions show the dynamics of CX3CR1-GFP^+/−^ labeled myeloid cells (green) captured in a wild type (left) vs. a IFN-I receptor deficient (right) OT-I CX3CR1-GFP^+/−^ mouse at day 15 post-infection. The maximal projections represent z-stacks (50 µm in depth) imaged over a 52 min time period. Blood vessels are shown in red. Note that microglia in the infected IFN-IR^−/−^ mouse remain non-activated and highly ramified when compared to the wild type control.
(MOV)

Table S1 **Differentially regulated genes during viral persistence.** Microarray analysis was performed on RNA extracted from the brains of LCMV-infected OT-I mice at days 15, 60, and 140 post-infection and compared to mock-infected mice (n = 3 mice per group). LCMV-infected OT-I mice were also compared to OT-I IFN-IR^−/−^ mice and corresponding mock-infected controls at days 5, 15, and 140 post-infection. Table S1 provides a list of the 604 genes that were differentially regulated in LCMV-infected OT-I and/or OT-I IFN-IR^−/−^ mice. The annotated data were used to generate the heat maps and interaction networks shown in Figures 2, 3, and 6. Fold-changes relative to mock-infected controls are shown for all three time points. Positive numbers indicate an increase in expression, whereas negative numbers represent decreased expression (relative to mock). The column entitled “Expression Pattern” is a binary representation of whether the genes are up (positive one), down (negative one), or unchanged (zero) in OT-I mice (days 5, 15, 60, 140 post-infection) or OT-I IFNR^−/−^ mice (days 5, 15, 140 post-infection). For example, “1111111” signifies that a particular gene was upregulated in both groups at all denoted time points. This binary representation was used to cluster genes into the categories shown in Figure 2B. The remaining columns represent pathways identified using AmiGO and IPA software (e.g. antigen presentation, cell death, chemokines, *etc.*) or genes included in the protein interaction network shown in Figure 2C. Boxes contain an “X” if a particular gene is considered part of the denoted pathway or network.
(XLSX)

Table S2 **Primers used for Q-PCR.** A list of primers and NCBI gene identification numbers is provided for all Q-PCR reactions performed in the study.
(DOC)
