# Supplementary figures and images for: Type I Interferon Programs Innate Myeloid Dynamics and Gene Expression in the Virally Infected Nervous System
Source: PLoS Pathog. 2013 May 30;9(5):e1003395. doi: 10.1371/journal.ppat.1003395 (PMC3667771; doi:10.1371/journal.ppat.1003395)

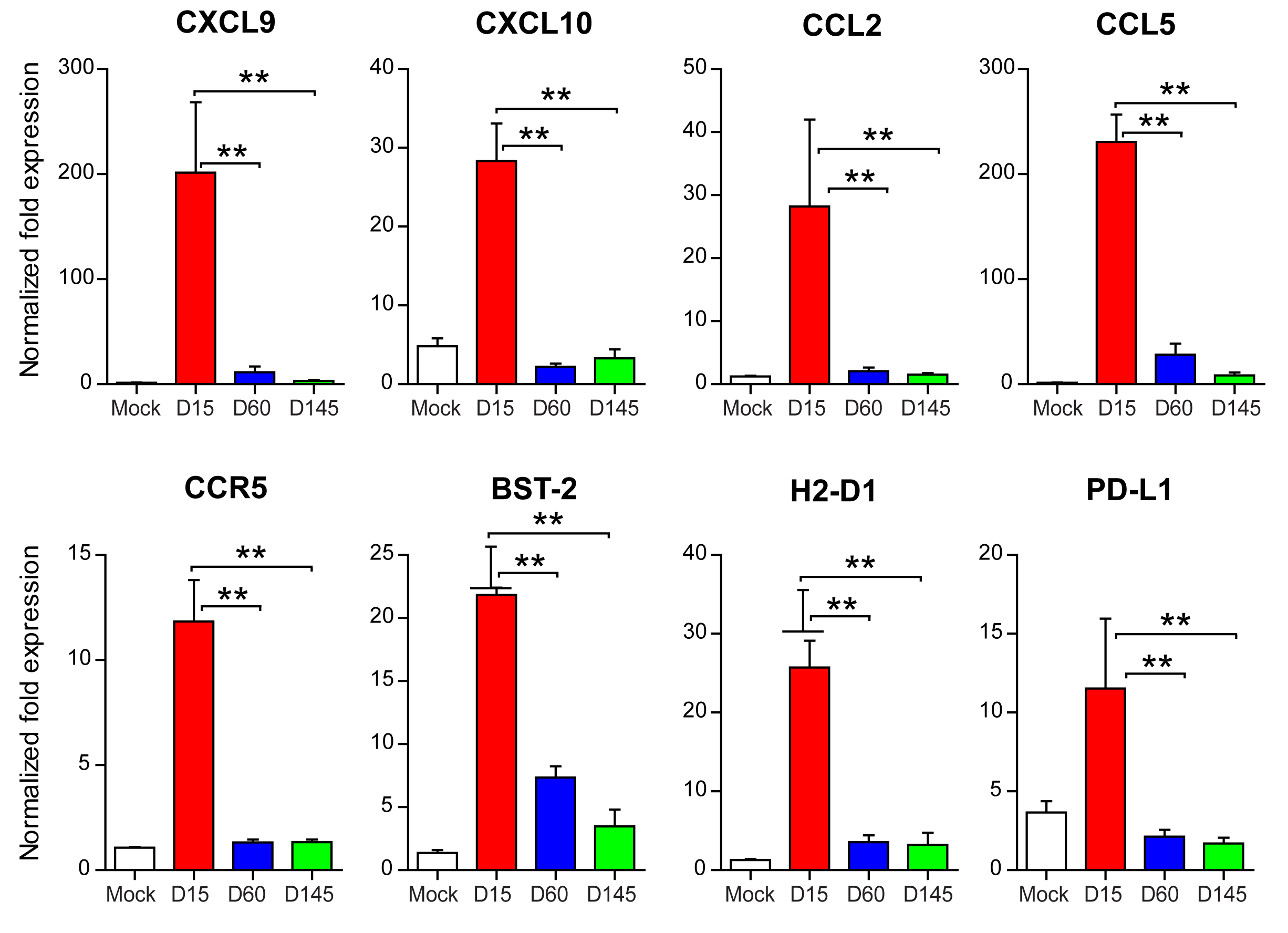

Supplement: Figure S1 — Validation of innate immune gene expression during viral persistence. Selected innate immune genes identified by microarray analysis (see Fig. 2 and Table S1) were quantified by Q-PCR using mRNA extracted from the brains of OT-I mice at days 15, 60, and 145 post-infection (n = 3 mice per group). Mock-infected mice were used as a control group. For all Q-PCR reactions, β-actin was used a reference gene to calculate normalized fold-expression. Data are plotted as mean ± SEM. Asterisks denote statistical significance (p<0.05). (TIF) [file ppat.1003395.s001.tif]

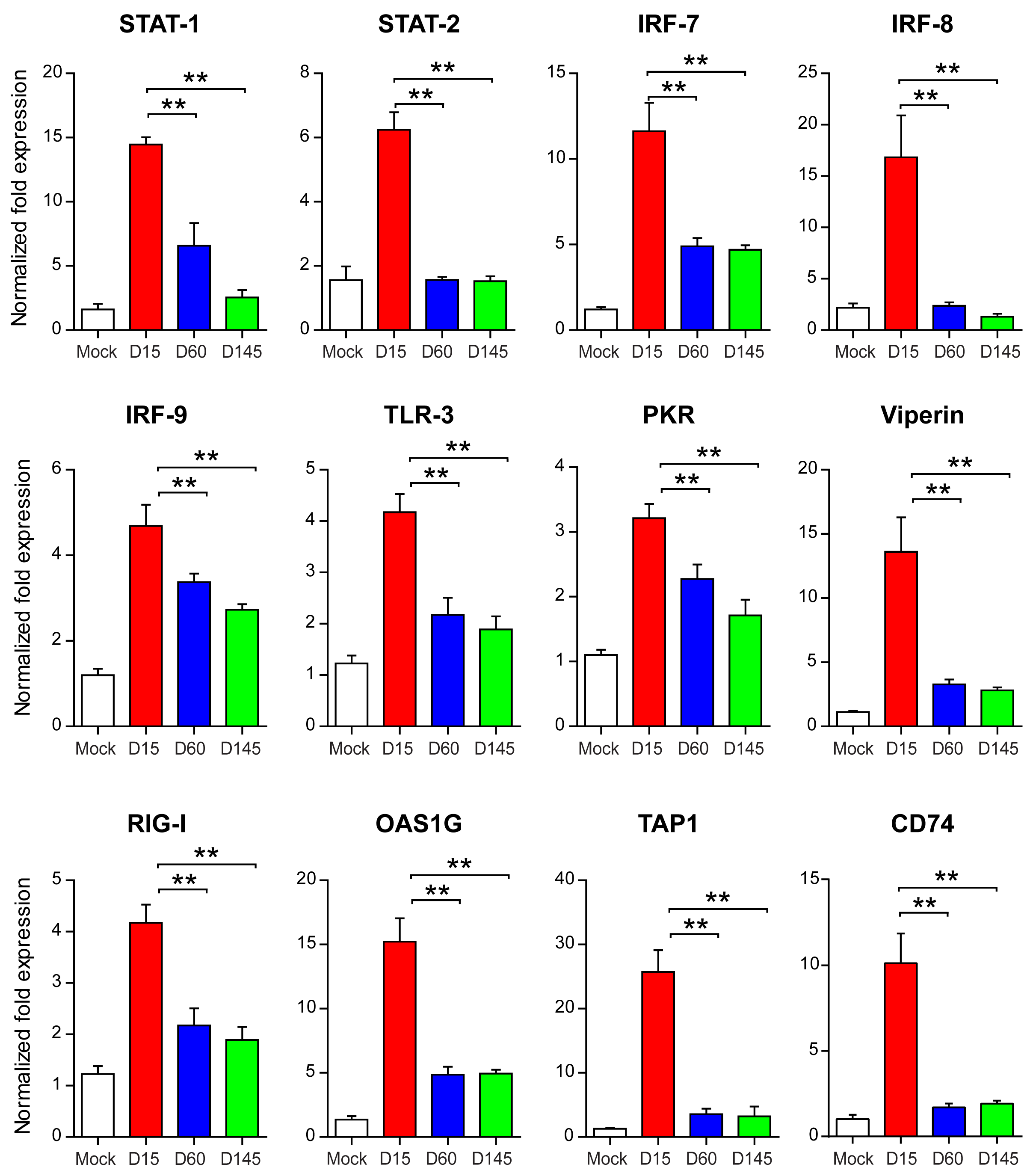

Supplement: Figure S2 — Validation of immune gene expression. Selected innate immune genes identified by microarray analysis (see Fig. 2 and Table S1) were quantified by Q-PCR using mRNA extracted from the brains of OT-I mice at days 15, 60, and 145 post-infection (n = 3 mice per group). β-actin was used a reference gene to calculate normalized fold-expression. Data are plotted as mean ± SEM, and asterisks denote statistical significance (p<0.05). (TIF) [file ppat.1003395.s002.tif]
