# Supplementary material for: Type I Interferon Programs Innate Myeloid Dynamics and Gene Expression in the Virally Infected Nervous System
Source: PLoS Pathog. 2013 May 30;9(5):e1003395. doi: 10.1371/journal.ppat.1003395 (PMC3667771; doi:10.1371/journal.ppat.1003395)
Supplement: Table S2 — Primers used for Q-PCR. A list of primers and NCBI gene identification numbers is provided for all Q-PCR reactions performed in the study. (DOC) [file ppat.1003395.s008.doc]

**Table S2. Q-PCR Primers**

**Gene NCBI ID Forward primer Reverse primer**

Actb NM_007393 GTGGGAATGGGTCAGAAG AGCTCATTGTAGAAGGTGTGG

BST2 NM_198095 CTCTTTCTATCACTATCTGCCC GGCGAAGTAGATTGTCAGGATG

CCL2 NM_011333 GTCCCTGTCATGCTTCTG GCTCTCCAGCCTACYTCATTG

CCL5 NM_013653 GGGTACCATGAAGATCTCTGC TCTAGGGAGAGGTAGGCAAAG

CCR5 NM_009917 TGTACAGCTCTCCTAGCCAG TCGGAACTGACCCTTGAAAATC

CXCL9 NM_008599 AGTCCGCTGTTCTTTTCCTC TGAGGTCTTTGAGGGATTTGTAG

CXCL10 NM_021274 TCAGCACCATGAACCCAA CTATGGCCCTCATTCTCACTG

H2D1 NM_010380 ATGAAGAGAAGGAGAAACACAGG TGAACACATCGTCTGTCACTC

IFNβ NM_010510 AAGAGTTACACTGCCTTTGCCATC CACTGTCTGCTGGTGGAGTTCATC

IFNα***** ATGGCTAGGCYCTGTGCTTTC TCTGAYCACCTCCCAGGCACA

IRF7 NM_001252601 TTGATCCGCATAAGGTGTACG TTCCCTATTTTCCGTGGCTG

IRF8 NM_008320 AGTTTACCGAATTGTCCCCG GTACTCATCCACAGAAGGTTCC

IRF9 NM_008394 CTCTTTGTTCAGCGCCTTTG GTACTGGGCCAAATCTCTACAG

LCMV-GP M20869.1 CATTCACCTGGACTTTGTCAGACTC GCAACTGCTGTGTTCCCGAAAC

OAS1G BC043339 AGAGATGCTTCCAAGGTGC ACTGATCCTCAAAGCTGGTG

PDL1 NM_021893 ACGTTTTGGAGATCACAGCC CAGCGTGGACACTACAATGAG

PKR NM_011163 TGGCTTAGGTGGATTTGGTC GTTGACGTGATTGAGTTCTGC

RIG-I NM_172689 GATGAAGGAGACAGAGAAGCTAG TCTGCCATCTGAAACACTGA

STAT-1 NM_009283 GCCGAGAACATACCAGAGAATC GATGTATCCAGTTCGCTTAGGG

STAT-2 NM_019963 AGAAGTCCTGCATTGGAGC ACCCTTATACCGAAGCATGTG

Tap1 NM_013683 CTCTTGGTGTTCATGTTTTGGG CGTGGACTTTGCTAGAGACTC

TLR3 NM_126166 CTTGGCTTTTGAGGTTGACG CCTTGCTTAGGTAACATCTATCCC

Viperin NM_021384 GAAACATTCTTGGAGCGTCA CAGAATAGACTTGGAAGGGTCC

Zbtb-16 NM_001033324 CCACCTTCGCTCACATACAG CACAGCCATTACACTCATAGGG

***** These are universal IFNα primers that detect all subtypes except IFNα4
